# Diagnostic and Prognostic Value of Lung Ultrasound B-Lines in Acute Heart Failure With Concomitant Pneumonia

**DOI:** 10.3389/fcvm.2021.693912

**Published:** 2021-08-19

**Authors:** Matteo Mazzola, Nicola Riccardo Pugliese, Martina Zavagli, Nicolò De Biase, Giulia Bandini, Giorgia Barbarisi, Gennaro D'Angelo, Michela Sollazzo, Chiara Piazzai, Simon David, Stefano Masi, Alberto Moggi-Pignone, Luna Gargani

**Affiliations:** ^1^Institute of Clinical Physiology, National Research Council, Pisa, Italy; ^2^Department of Clinical and Experimental Medicine, University of Pisa, Pisa, Italy; ^3^Department of Experimental and Clinical Medicine, Azienda Ospedaliera Universitaria Careggi, Florence, Italy; ^4^Sheba Medical Center, Ramat Gan, Israel

**Keywords:** lung ultrasound, B-lines, pulmonary congestion, acute heart failure, pneumonia, prognosis

## Abstract

**Purpose:** To evaluate the potential confounding effect of concomitant pneumonia (PNM) on lung ultrasound (LUS) B-lines in acute heart failure (AHF).

**Methods:** We enrolled 86 AHF patients with (31 pts, AHF/PNM) and without (55 pts, AHF) concomitant PNM. LUS B-lines were evaluated using a combined antero-lateral (AL) and posterior (POST) approach at admission (T0), after 24 h from T0 (T1), after 48 h from T0 (T2) and before discharge (T3). B-lines score was calculated at each time point on AL and POST chest, dividing the number of B-lines by the number of explorable scanning sites. The decongestion rate (DR) was calculated as the difference between the absolute B-lines number at discharge and admission, divided by the number of days of hospitalization. Patients were followed-up and hospital readmission for AHF was considered as adverse outcome.

**Results:** At admission, AHF/PNM patients showed no difference in AL B-lines score compared with AHF patients [AHF/PNM: 2.00 (IQR: 1.44–2.94) vs. AHF: 1.65 (IQR: 0.50–2.66), *p* = 0.072], whereas POST B-lines score was higher [AHF/PNM: 3.76 (IQR: 2.70–4.77) vs. AHF = 2.44 (IQR: 1.20–3.60), *p* < 0.0001]. At discharge, AL B-lines score [HR: 1.907 (1.097–3.313), *p* = 0.022] and not POST B-lines score was found to predict adverse events (AHF rehospitalization) after a median follow-up of 96 days (IQR: 30–265) in the overall population.

**Conclusions:** Assessing AL B-lines alone is adequate for diagnosis, pulmonary congestion (PC) monitoring and prognostic stratification in AHF patients, despite concomitant PNM.

## Introduction

Concomitant pneumonia (PNM) is commonly observed in elderly patients admitted for acute heart failure (AHF) to Internal Medicine Departments (1, 2) with a high prevalence of comorbidities (e.g., diabetes mellitus and COPD). This condition can represent a precipitating factor or a subsequent complication of AHF with a bidirectional causality link, and is independently associated with in-hospital mortality (1, 3). As rales and dyspnea represent cardinal signs and symptoms of both diseases, the clinical diagnosis of AHF/PNM association is usually challenging, especially in the elderly population that displays less often respiratory and non-respiratory symptoms of PNM (1, 4, 5). The presence of infiltrates demonstrated by imaging is indeed mandatory for PNM diagnosis according to the current guidelines (6, 7). Lung ultrasound (LUS) has demonstrated high sensibility and specificity in PNM, allowing the identification of parenchymal consolidations (8–10). On the other hand, being an indirect effect of the increase in extravascular lung water (EVLW), LUS B-lines provide the clinician with an accurate, non-invasive and low-cost technique for pulmonary congestion (PC) evaluation in AHF patients. Substantial evidence supports this echographic approach as a useful diagnostic tool and valid prognosticator in emergency departments and outpatient clinics (11–19). In patients admitted for AHF, LUS B-lines evaluation at discharge can detect sub-clinical residual PC, which proved to predict adverse outcome (e.g., hospitalization for worsening HF) for up to 6 months (12–14). Presence of PNM in AHF patients could potentially increase LUS B-lines as a result of the combination between cardiogenic oedema and inflammatory oedema, but up to date, little is known about this topic. This study aimed to evaluate the potential confounding effect of PNM on LUS evaluation of B-lines in AHF patients. In addition to the traditional antero-lateral (AL) chest approach, we also performed posterior (POST) chest LUS, which is usually assessed in patients with non-cardiogenic B-lines (e.g., in pulmonary fibrosis) (20, 21) and/or suspected consolidations (e.g., PNM) (8–10). Furthermore, in critically-ill patients in intensive care units with acute lung injury/acute respiratory distress syndrome (ALI/ARDS), the postero-lateral chest is also usually scanned, whenever possible (22). In previous studies on LUS B-lines in HF management, only AL areas have been taken into account as scanning sites and, to the best of our knowledge, this is the first study to include a comprehensive AL and POST B-lines evaluation in AHF patients.

## Materials and Methods

### Patients Population

We conducted a prospective, monocentric, observational study in adults hospitalized for AHF, regardless of left ventricular ejection fraction (LVEF). Patients were recruited from the Internal Medicine Department of Careggi University Hospital in Florence. AHF diagnosis was based on the 2016 European Guidelines (23). Patients were subdivided, according to LVEF, in heart failure with reduced ejection fraction (HFrEF) and heart failure with preserved ejection fraction (HFpEF) (23). We included AHF patients diagnosed with concomitant PNM according to current recommendations for diagnosis and management of community-acquired PNM in adults (6). Patients were furthermore sub-classified, according to the presence of concomitant PNM, in AHF and AHF/PNM. We also included in the analysis 25 patients with a diagnosis of PNM, according to current recommendations (6), without AHF (PNM group).

Exclusion criteria were: the onset of AHF in the clinical context of an acute coronary syndrome (ACS), a moderate-to-severe interstitial chronic lung disease defined by pulmonary function tests and/or computed tomography scans (pulmonary fibrosis or known pulmonary malignancy) to avoid potential bias in LUS findings, dialysis, pregnancy and NT-proBNP below the age-adjusted cut point in the presence of LVEF >50% (≤ 900 pg/mL ages 50–75; ≤ 1,800 pg/mL over age 75) (24). Patients with chronic obstructive pulmonary disease were not excluded from the study population. The local Ethical Committee approved the study. All subjects gave informed consent, and the study was performed in accordance with the ethical standards of the 1964 Helsinki declaration and its later amendments, with local guidelines for good clinical practice.

### Lung Ultrasound

Each patient underwent a complete LUS examination of AL and POST scanning sites at admission (T0), performed by a trained investigator (25, 26). AL evaluation was performed with the patient in a recumbent or semi-recumbent position, using a standard imaging protocol consisting of 28 scanning sites (25, 26). Conversely, for POST evaluation, patients were asked to stay in a seated position with their back facing the operator, and a 32-scanning sites scheme was performed as previously described (21). The complete examination was repeated, as per protocol: within 24 h from T0 (T1), after 48 h from T0 (T2) and before hospital discharge (T3). LUS B-lines have been quantified as previously described: in clearly distinguishable B-lines, a one-by-one count was performed; for confluent B-lines, we visually estimated the percentage of hyperechogenicity (“white” screen below the pleural line) generated by B-lines, and the number of B-lines was estimated dividing this value by 10 (i.e., 70% of white screen below the pleural line equals to about 7 B-lines) (27). In order to correct for the higher number of scanning sites in POST chest, a standardized B-lines score was calculated at each time point on AL and POST chest dividing the number of B-lines by the number of explorable scanning sites. The decongestion rate (DR) was calculated as the difference between the absolute B-lines number at discharge and at admission, divided by the number of days of hospitalization. The LUS inter-observer variability was examined by intraclass correlation coefficient (ICCs) before the enrolment on 50 previously acquired LUS videos evaluated by an expert reader (L.G.), using a standardized training protocol (28). The mean ICC on B-lines number assessment was 0.962 (single measurements, *p* < 0.0001) and 0.981 (average measurements, *p* < 0.0001) between the expert reader and reader 1 (G.B.), consistent with previous data (28).

### Clinical and Follow-Up Data

Clinical and demographic data were taken from medical records. Follow-up data were obtained in all enrolled patients through phone calls, review of electronic medical records or by contacting primary care physicians or cardiologists. We considered rehospitalisation for AHF as an adverse outcome. The event was defined according to European Guidelines for Acute and Chronic HF diagnosis and treatment (23).

### Statistical Analysis

Data were analyzed with SPSS version 25.0 (IBM Corp., Armonk, NY). Continuous measures were expressed as mean value ± standard deviation or median and interquartile range, as appropriate. Categorical variables were presented as percentages. Mann-Whitney test and Kruskal-Wallis one way ANOVA test were used to assess the differential distribution of data among samples. Cox proportional hazard regression analysis was used to identify outcome predictors. We excluded collinearity using variance inflation factor. A *p*-value of 0.05 was used as cut-off to determinate statistical significance. To achieve an alpha value of 0.05 and a beta value of 0.8 to establish a significant difference in the number of B-lines on AL chest between AHF and AHF/PNM groups, we calculated that a total sample size of 66 patients was needed (88 patients for a beta value of 0.9).

## Results

**Figure 1:**
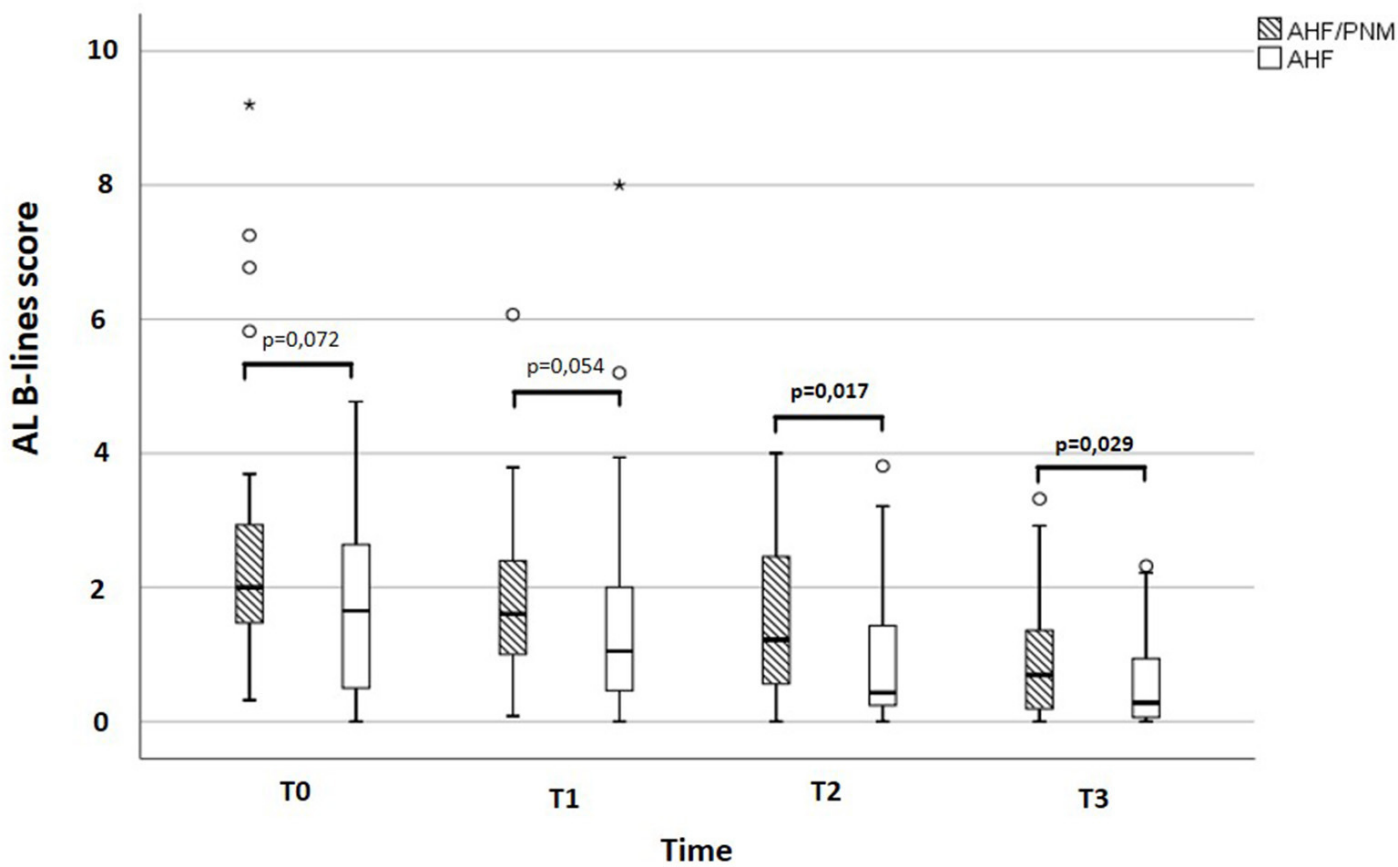
Difference in AL B-lines score at each time point between AHF/PNM and AHF patients. Box and whisker graph describing the difference in AL B-lines score between AHF/PNM and AHF patients. POST, posterior; AHF, isolated acute heart failure; AHF/PNM, acute heart failure with concomitant pneumonia; PNM, pneumonia.

**Figure 2:**
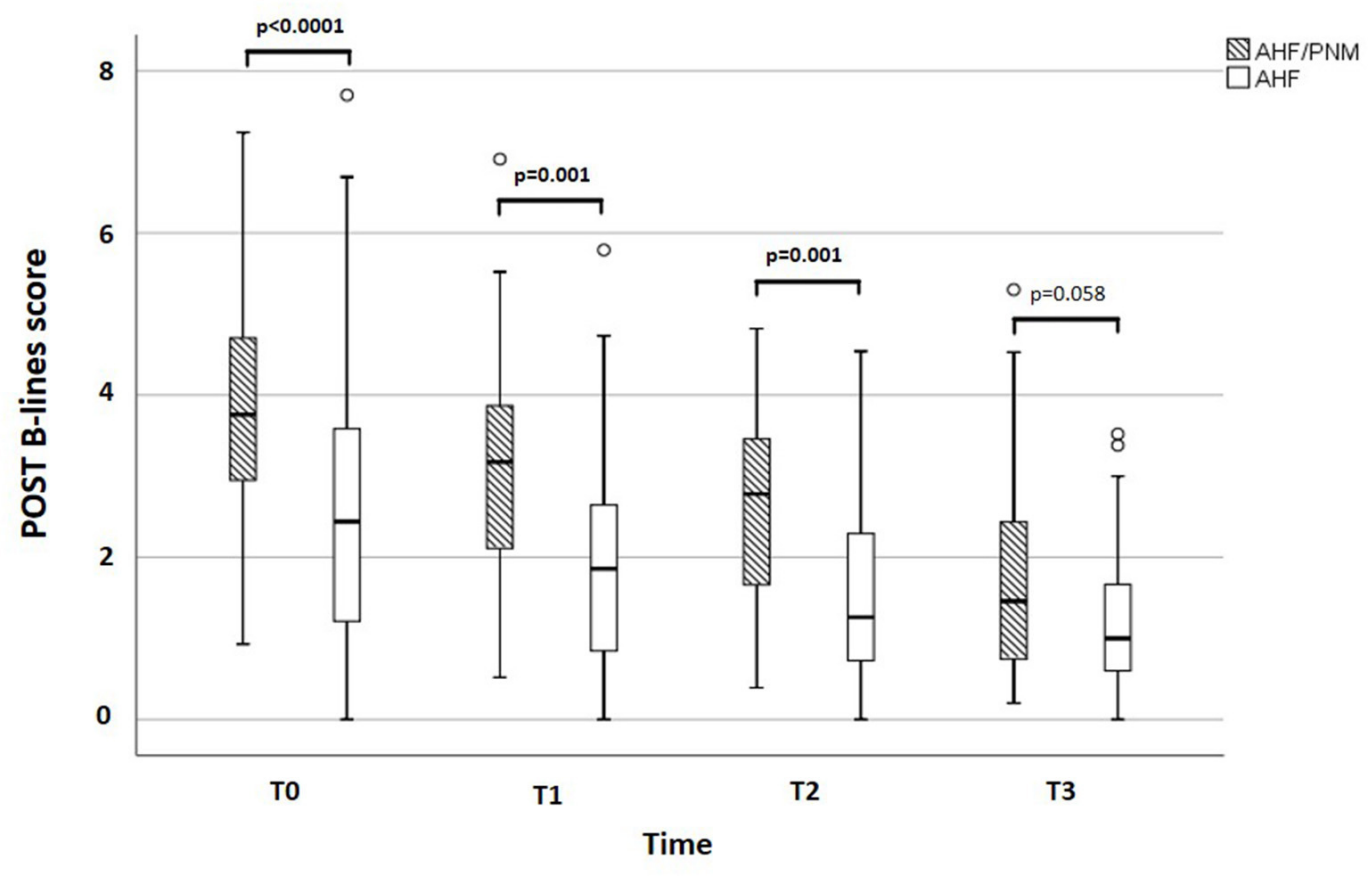
Difference in POST B-lines score at each time point between AHF/PNM and AHF patients. Box and whisker graph describing the difference in POST B-lines score between AHF/PNM and AHF patients. POST, posterior; AHF: isolated acute heart failure; AHF/PNM, acute heart failure with concomitant pneumonia; PNM, pneumonia.

**Figure 3:**
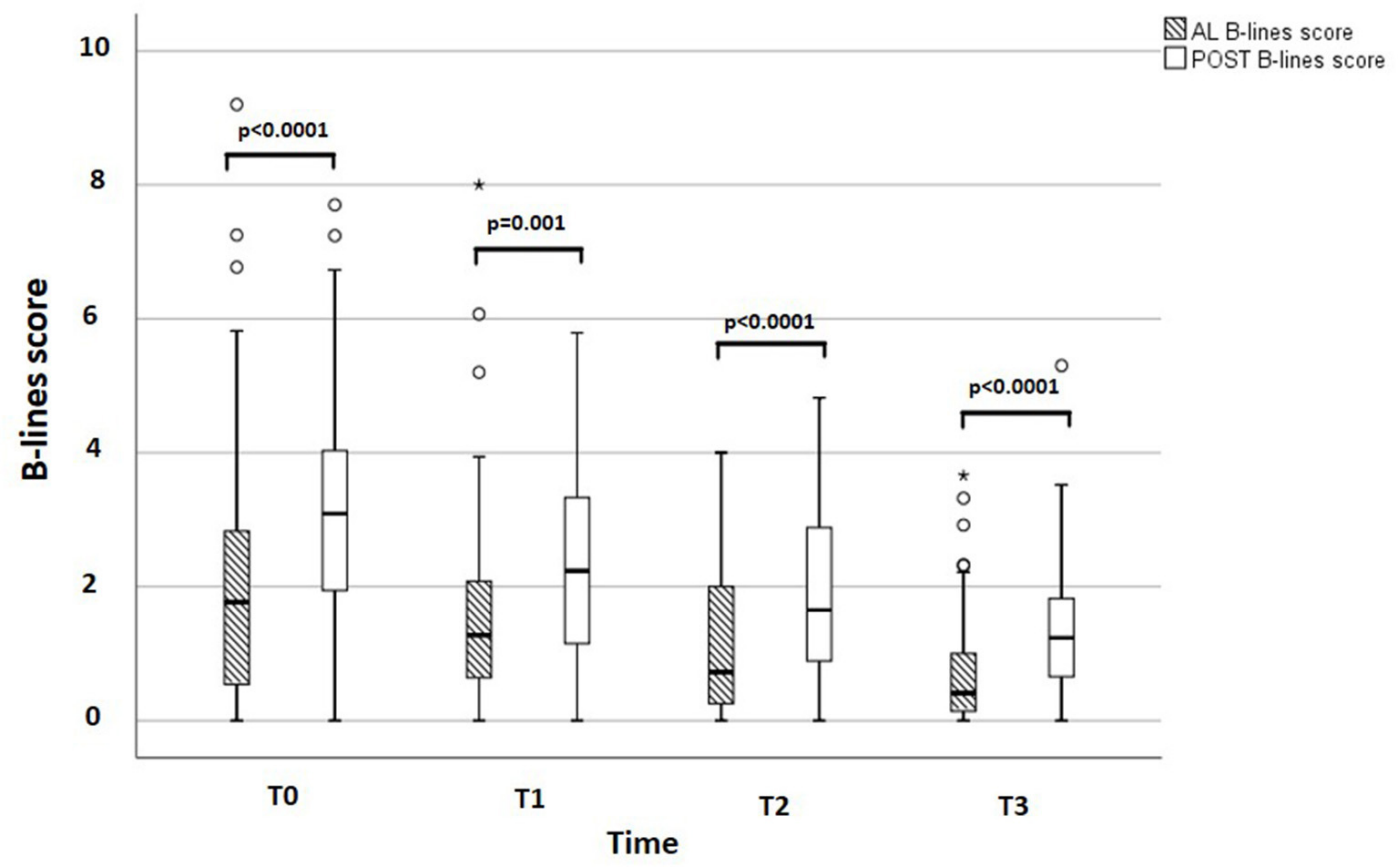
AL and POST B-lines score at each time point. Box and whisker graph describing AL and POST B-lines score at each time point. AL, antero-lateral; POST, posterior.

We enrolled a total of 86 consecutive AHF patients: median age 84 (IQR: 79–89) years, 46 (53%) females. Fifty-five patients (64%) had AHF, and 31 (36%) had AHF/PNM. Thirty-nine patients (45%) had HFrEF (45%) and 47 (55%) had HFpEF. The main characteristics of the study population, including demographics, clinical and bio-humoral data, are reported in Table 1. Compared to patients with AHF, AHF/PNM patients were more commonly former or actual smoker and displayed a higher prevalence of COPD (Table 1). Compared with AHF patients, no difference in AL B-lines score was observed in AHF/PNM patients at admission (T0) [AHF/PNM: 2.00 (IQR: 1.44–2.94) vs. AHF: 1.65 (IQR: 0.50–2.66), *p* = 0.072]. Conversely, at discharge (T3), AHF/PNM patients displayed a slightly higher score [AHF/PNM: 0.70 (IQR: 0.19–1.41) vs. AHF: 0.28 (IQR: 0.04–0.96), *p* = 0.029] (Table 2, Figure 1). Regarding POST B-lines score, the value at admission (T0) was higher compared to AHF patients [AHF/PNM: 3.76 (IQR: 2.70–4.77) vs. AHF = 2.44 (IQR: 1.20–3.60), *p* < 0.0001], as well as at T1 and T2, whereas no difference was noted at discharge [AHF/PNM: 1.46 (IQR: 0.73–2.47) vs. AHF: 1.00 (IQR: 0.60–1.70), *p* = 0.058] (Table 2, Figure 2). In the overall population, the absolute number of B-lines was higher on POST chest compared to AL chest at all time-points [B-lines AL T0: 37 (IQR: 13.5–60.5) vs. B-lines POST T0: 62 (IQR: 35–96), *p* < 0.0001; B-lines AL T1: 32 (IQR: 14–50) vs. B-lines POST T1: 44.5 (IQR: 21–76), *p* = 0.007; B-lines AL T2: 15 (IQR: 7–40) vs. B-lines POST T2: 36 (IQR: 14–61.3), *p* < 0.0001; B-lines AL T3: 8 (IQR: 3–20) vs. B-lines POST T3: 26 (IQR: 15.5–46.5), *p* < 0.0001]. Comparing AL and POST B-lines score, we observed that, even after indexing the number of B-lines for the number of scanning sites, POST scanning sites displayed a higher number of B-lines than AL [score AL T0: 1.80 (IQR: 0.59–2.79) vs. score POST T0: 3.08 (IQR: 1.94–4.03), *p* < 0.0001; score AL T1: 1.32 (IQR: 0.64–2.08) vs. score POST T1: 2.23 (IQR: 1.15–3.34), *p* = 0.001; score AL T2: 0.75 (IQR: 0.25–2.00) vs. score POST T2: 1.65 (IQR: 0.87–2.92), *p* < 0.0001; score AL T3: 0.40 (IQR: 0.13–1.00) vs. score POST T3: 1.21 (IQR: 0.60–1.85), *p* < 0.0001] (Table 3, Figure 3). When considering patients according to LVEF, no difference was found either in AL or in POST B-lines score at each time point (Supplementary Table 1). To further investigate the effect of PNM on AL and POST B-lines, we also enrolled 25 patients with a primary diagnosis of PNM without AHF (PNM group). Compared to AHF and AHF/PNM groups, PNM patients displayed the lowest values of AL and POST B-lines score (Supplementary Table 2). The comparison among the three groups demonstrated that the presence of PNM significantly affects only POST B-lines score at admission in AHF/PNM patients (Supplementary Table 2). Conversely, AL B-lines score at both admission and discharge and AL decongestion rates did not show any significant difference between AHF and AHF/PNM groups (Supplementary Table 2). Patients with AHF/PNM showed the highest POST decongestion rates compared to the other groups. We then stratified the analysis of left and right B-lines score according to the site of PNM (Supplementary Table 3). Almost 40% of our patients had a bilateral PNM and the site of PNM didn't affect left and right B-lines scores (Supplementary Table 3).

**Table 1:** Patient clinical characteristics in the overall population, AHF and AHF/PNM groups.

**Variable**	**Total population**	**AHF**	**AHF/PNM**	**p**
	**(*n* = 86)**	**(*n* = 55)**	**(*n* = 31)**	
**Demographics**				
Age, years	84 (79–89)	84 (79–89)	83 (78–87)	0.26
Female gender	46 (53)	32 (58)	14 (45)	0.31
Family history of CAD	7 (8)	4 (7)	3 (10)	0.66
Diabetes mellitus	27 (31)	15 (27)	12 (39)	0.23
Arterial hypertension	74 (86)	48 (87)	26 (84)	0.62
Dyslipidaemia^∧^	26 (30)	16 (29)	10 (32)	0.97
Smoking	41 (47)	20 (36)	21 (68)	**0.003**
CAD	31 (36)	19 (35)	12 (39)	0.85
Previous MI	30 (35)	19 (35)	11 (35)	0.91
Atrial fibrillation	53 (62)	33 (60)	20 (65)	0.54
COPD	22 (26)	9 (16)	13 (42)	**0.007**
**In-hospital evaluation (admission)**				
Hb (g/dL)	11.8 (10.4–13.5)	12.3 (10.2–13.5)	11.2 (10.4–12.7)	0.48
Creatinine (mg/dL)	1.26 (0.97–1.50)	1.35 (0.90–1.50)	1.20 (1.00–1.50)	0.89
eGFR (mL/min/1.73m^2^)	45 (36–68)	44 (35–68)	45 (37–65)	0.67
NT-proBNP (pg/mL)	7,073 (3,843–10,936)	7,087 (2,686–11,116)	6,840 (4,151–11,532)	0.61
LVEF (%)	50 (35–57)	46 (35–56)	48 (35–56)	0.78
Pleural effusion	49 (57)	30 (55)	19 (60)	0.43

*Data are presented as n (%), mean and 95% confidence interval if normally distributed, or median and first and third quartile if not normally distributed*.

∧ *total cholesterol ≥200 mg/dL or LDL-C ≥130 mg/dL or lipid-lowering therapy*.

*CAD, coronary artery disease; Hb, hemoglobin; eGFR, estimated glomerular filtration rate; MI, myocardial infarction; NT-proBNP, N-terminal prohormone of brain natriuretic peptide; LVEF, left ventricular ejection fraction; COPD, chronic obstructive pulmonary disease. Statistically significant p-values (p < 0.05) are in bold*.

**Table 2:** Difference in AL and POST B-lines score between AHF and AHF/PNM at each time point.

**Time**	**AHF (*n* = 55)**	**AHF/PNM (*n* = 31)**	***p***
**AL**			
T0	1.65 (0.50–2.66)	2.00 (1.44–2.94)	0.072
T1	1.05 (0.46–2.00)	1.61 (0.94–2.49)	0.054
T2	0.43 (0.21–1.47)	1.22 (0.56–2.47)	**0.017**
T3	0.28 (0.04–0.96)	0.70 (0.19–1.41)	**0.029**
**POST**			
T0	2.44 (1.20–3.60)	3.76 (2.70–4.77)	**<0.0001**
T1	1.86 (0.77–2.67)	3.18 (2.06–3.91)	**0.001**
T2	1.26 (0.72–2.31)	2.78 (1.64–3.49)	**0.001**
T3	1.00 (0.60–1.70)	1.46 (0.73–2.47)	0.058

*Data are presented as n (%), mean and 95% confidence interval if normally distributed, or median and first and third quartile if not normally distributed*.

*POST, posterior; AHF, acute heart failure; PNM, pneumonia. T0, admission; T1, 24 h from admission; T2, 48 h from admission; T3, discharge. Statistically significant p-values (p < 0.05) are in bold*.

**Table 3:** AL and POST B-lines score at each time point.

**Time**	**AL B-Lines Scores**	**POST B-Lines Score**	***P***
T0	1.80 (0.59–2.70)	3.08 (1.94–4.03)	**<0.0001**
T1	1.32 (0.64–2.08)	2.23 (1.15–3.34)	**0.001**
T2	0.75 (0.25–2.00)	1.65 (0.87–2.92)	**<0.0001**
T3	0.40 (0.13–1.00)	1.21 (0.60–1.85)	**<0.0001**

*Data are presented as n (%), mean and 95% confidence interval if normally distributed, or median and first and third quartile if not normally distributed*.

*AL,antero-lateral; POST, posterior. T0, admission; T1, 24 h from admission; T2, 48 h from admission; T3, discharge. Statistically significant p-values (p < 0.05) are in bold*.

During monitoring between admission and discharge, AL and POST chest DR were comparable [DR-AL: −3.17 (IQR: −6.63 to −1.27) vs. DR-POST: −5.07 (−7.3 to −1.17), *p* = 0.167]. During follow-up (median length: 96 days; IQR: 30–265), 12 readmissions for AHF occurred. Only AL B-lines score at discharge and creatinine levels were found to predict adverse events at univariate and multivariate analysis [HR AL B-Lines score T3: 2.95 (1.21–7.18), *p* = 0.02; HR creatinine: 9.1 (1.67–49.6), *p* = 0.01] (Table 4).

**Table 4:** Univariate analysis of AHF hospitalization during follow-up (median length: 96 days; IQR: 30–265).

**Parameter**	**Overall population (*n* = 86)**	
	**HR (95% CI)**	***p*-value**
**Univariate**		
AL B-lines score T3	**1.907 (1.097–3.313)**	**0.022**
POST B-lines score T3	1.14 (0.688–1.913)	0.59
AL B-lines score T0	1.098 (0.749–1.609)	0.63
POST B-lines score T0	0.878 (0.617–1.250)	0.47
POST DR	1.092 (0.954–1.251)	0.20
AL DR	1.090 (0.921–1.290)	0.32
Age (0,1)	1.02 (0.92–1.14)	0.67
Sex (0,1)	0.015 (0.00–10.8)	0.21
NTproBNP at admission (pg/mL)	(1.00–1.00)	0.15
LVFE (%)	0.97 (0.88–1.07)	0.63
Creatinine (mg/dL)	**12.6 (2.8–56.3)**	**<0.001**
**Multivariate**		
AL B-lines score T3	**2.95 (1.21–7.18)**	**0.02**
Creatinine (mg/dL)	**9.1 (1.67–49.6)**	**0.01**

*AHF, acute heart failure; AL, antero-lateral; POST, posterior; DR, decongestion rate; T0, admission; T3, discharge. Statistically significant p-values (p < 0.05) are in bold*.

## Discussion

We found that although POST B-lines score is different at admission between AHF and AHF/PNM patients, AL B-lines score is not significantly different in the two populations. Moreover, during monitoring DR were similar on AL and POST chest, and at follow-up, only AL B-lines score and not POST B-lines score is able to predict rehospitalization for AHF at 3 months. Overall, these data suggest that a limited AL sonographic evaluation of the chest is enough for the diagnosis, monitoring and prognostic stratification of AHF, and that LUS value in AHF assessment is valid regardless of the presence of concomitant pneumonia. Patients with HF display a higher risk of PNM, as the increase in EVLW reduces microbial lung clearance (5, 29). On the other hand, PNM affects the cardiovascular system at different levels. Non-ischemic myocardial injury is observed as a direct effect of the pathogen and/or as a result of high levels of circulating inflammatory cytokines (30). The effect of systemic inflammation is further related to both endothelial dysfunction and acute kidney injury, causing an increase in afterload and preload, respectively (30). A concomitant PNM represents a possible confounding factor for LUS evaluation of PC in AHF patients due to the association of hemodynamic and inflammatory oedema. PC is one of the main features of patients with HF and the main pathophysiological reason of AHF hospitalizations and readmissions (31–33). The sensibility and specificity of LUS B-lines in detecting PC support the use of B-lines as “point-of-care” ultrasound approach in different relevant settings, from emergency departments to outpatients clinics, for the differential diagnosis of dyspnea of unclear origin, to rule in or rule out AHF (11–19). According to our results, there is no difference in AL B-lines score at admission between AHF and AHF/PNM group. Therefore, the presence of PNM does not seem to significantly affect AL B-lines evaluation for AHF diagnosis. Conversely, AHF/PNM displayed higher POST B-lines score at admission compared to AHF patients, likely as a direct confounding effect of PNM, which is indeed more frequently located in the posterior chest. We evaluated B-lines at different time points during AHF hospitalization, to check the potential confounding effect of concomitant PNM on the decrease in B-lines number. We found no significant difference in the DR between AL and POST chest, thus confirming that LUS is able to monitor pulmonary decongestion in both populations, irrespective of the presence of PNM. This can be relevant in therapy titration, especially to monitor the effects of diuretics which are the cornerstone of AHF treatment, but should be used with caution especially in older patients with comorbidities and pulmonary conditions. Even when introducing a “control” group of patients with only PNM, B-lines scores are not significantly different at admission on the AL chest in patients with AHF/PNM compared to patients with only AHF, whereas they are significantly increased on the POST chest. Therefore, LUS can be used to diagnose AHF also in patients with concomitant PNM, because the AL picture at admission is not significantly different in terms of B-lines; only the assessment of the POST chest would introduce an additional number of B-lines, likely due to the inflammatory oedema.

Concerning prognostic stratification, up to 50% patients admitted with AHF are discharged with residual PC, which in turn is associated with an increased risk of rehospitalization and death within 6 months (12–14). Clinical evaluation and other non-invasive tools display a low sensitivity and poor predictive value (34, 35) and the evidence supporting the role of B-lines evaluation in monitoring AHF therapy has been increasing. Indeed, we observed that AL B-lines score at discharge was able to predict AHF rehospitalization in patients with and without PNM. Interestingly, AHF/PNM patients displayed higher AL B-lines score at discharge compared to AHF patients. This may be related to the inhibition of the hypoxia-induced pulmonary vasoconstriction (HPV) that has been observed in animal models with PNM (36, 37), which can in part limit the effect of diuretics (38). However, the difference we observed between AHF and AHF/PNM patients was not associated with a different outcome (AHF re-hospitalization) during the follow-up. Therefore, it might be conceivable that the discharge difference in AL B-lines was too small to maintain a significant impact on prognosis later on. The current pandemic caused by severe acute respiratory syndrome coronavirus 2 (SARS-CoV-2) can represent a further pathophysiological model to test this hypothesis and confirm our findings.

There are some limitations to be acknowledged. The sample size is relatively small, and the study was conducted in a single center. For AL chest evaluation, we used a 28 scanning sites imaging protocol, which is more time-consuming than the simplified protocols involving 4 or 8 scanning sites (12, 39). The protocol was not designed to evaluate pulmonary consolidations that are the main LUS sign to rule in PNM, given the large amount of literature on this topic, and PNM was not defined according to LUS, therefore we reported only data about B-lines. We did not report any other echocardiographic parameter than LVEF because data were not available in the whole population. LUS operators were not completely blinded to the group stratification, although the final correct diagnosis was adjudicated only at the end of the hospitalization, whereas LUS exams were performed at admission, when there could have been only a clinical suspicion for a certain condition.

Our findings confirm the role of LUS B-lines evaluation in the management of AHF patients, and suggest that an approach limited to AL scanning sites can be sufficient both in the diagnosis and risk stratification of AHF patients during hospitalization, despite the presence of concomitant PNM.

## Data Availability Statement

The raw data supporting the conclusions of this article will be made available by the authors, without undue reservation.

## Ethics Statement

The studies involving human participants were reviewed and approved by Comitato Etico Regionale per la Sperimentazione Clinica della Regione Toscana, Sezione AREA VASTA CENTRO. The patients/participants provided their written informed consent to participate in this study.

## Author Contributions

MM contributed to the conception of the study, data analysis, work drafting, gave final approval of the version to be published, and is the guarantor of the paper. NP contributed to the data analysis, work drafting, and gave final approval of the version to be published. MZ, GBan, GBar, and MS contributed to the data acquisition and gave final approval of the version to be published. ND contributed to the conception of the study, work drafting, and gave final approval of the version to be published. GD'A, SM, and AM-P contributed to the conception of the study and gave final approval of the version to be published. LG contributed to the conception of the study, data analysis, work drafting, and gave final approval of the version to be published. All authors contributed to the article and approved the submitted version.

## Conflict of Interest

The authors declare that the research was conducted in the absence of any commercial or financial relationships that could be construed as a potential conflict of interest.

## Publisher's Note

All claims expressed in this article are solely those of the authors and do not necessarily represent those of their affiliated organizations, or those of the publisher, the editors and the reviewers. Any product that may be evaluated in this article, or claim that may be made by its manufacturer, is not guaranteed or endorsed by the publisher.
