# Supplementary material for: Diagnostic and Prognostic Value of Lung Ultrasound B-Lines in Acute Heart Failure With Concomitant Pneumonia
Source: Front Cardiovasc Med. 2021 Aug 19;8:693912. doi: 10.3389/fcvm.2021.693912 (PMC8416771; doi:10.3389/fcvm.2021.693912)
Supplement: Supplementary file 1 [file Data_Sheet_1.docx]

| Time | HFrEF | HFpEF | p |
| --- | --- | --- | --- |
|  |  |  |  |
| AL |  |  |  |
| T0 | 1.80 (0.46-2.64) | 1.79 (0.78-2.87) | 0.57 |
|  |  |  |  |
|  |  |  |  |
| T1 | 1.57 (0.68-2.46) | 1.16 (0.60-2.02) | 0.56 |
|  |  |  |  |
|  |  |  |  |
| T2 | 0.73 (0.23-1.70) | 0.78 (0.29-2.06) | 0.58 |
|  |  |  |  |
|  |  |  |  |
| T3 | 0.36 (0.058-0.95) | 0.40 (0.14-1.14) | 0.44 |
|  |  |  |  |
|  |  |  |  |
| POST |  |  |  |
| T0 | 2.81 (1.85-4.04) | 3.19 (1.82-4.02) | 0.76 |
|  |  |  |  |
|  |  |  |  |
| T1 | 2.18 (1.04-3.81) | 2.25 (1.28-3.20) | 0.73 |
|  |  |  |  |
| T2 | 1.69 (0.92-3.08) | 1.64 (0.64-2.77) | 0.62 |
|  |  |  |  |
|  |  |  |  |
| T3 | 1.26 (0.63-2.26) | 1.03 (0.59-1.64) | 0.27 |
|  |  |  |  |
|  |  |  |  |

**Supplemental Table 1.** Difference in AL and POST B-Lines Score between HFrEF and HFpEF at each time point

Data are presented as n (%), mean and 95% confidence interval if normally distributed, or median and first and third quartile if not normally distributed.

POST: posterior; HFrEF: heart failure with reduced ejection fraction; HFpEF: heart failure with preserved ejection fraction. T0: admission; T1: 24h from admission; T2: 48h from admission; T3: discharge.

**Supplemental Table 2.** Differences in demographic characteristics and LUS among AHF, AHF/PNM and PNM groups

|  | AHF  (n=55) | AHF/PNM  (n=31) | PNM  (n=25) | p |
| --- | --- | --- | --- | --- |
| Demographics |  |  |  |  |
| Age, years | 84 (79-89) | 83 (78 – 87) | 79 (77 – 84) | 0.07 |
| Female gender | 32 (58) | 14 (45) | 13 (52) | 0.39 |
| LUS |  |  |  |  |
| AL score T0 | 1.65 (0.50-2.66)* | 2.00 (1.44-2.94)* | 0.14 (0.11 – 0.23) | **p<0.001** |
| AL score T3 | 0.28 (0.04-0.96)* | 0.70 (0.19-1.41)* | 0.07 (0.00 – 0.11) | **p<0.001** |
| POST score T0  POST score T3  AL decongestion rate  POST decongestion rate | 2.44 (1.20-3.60)*  1.00 (0.60-1.70)*  - 2.00 (-0.1 - - 5.6)*  - 1.25 (- 6.3 - -0.13) | 3.76 (2.70-4.77)*°  1.46 (0.73-2.47)*  -3.7 (-1.4 - - 7.4)*  - 5.4 (-7.3 - - 3.00)*° | 0.56 (0.28 – 0.86)   - 1. (0.00 – 0.27)   -0.16 (-0.22 - - 1.3)  -0.71 (- 1.1 - - 0.28) | **p<0.001**  **p<0.001**  **p<0.001**  **p<0.001** |

Data are presented as n (%), mean and 95% confidence interval if normally distributed, or median and first and third quartile if not normally distributed.

AL: antero-lateral; POST: posterior; AHF: acute heart failure; PNM: patients with only pneumonia; AHF/PNM: patients with acute heart failure and concomitant pneumonia.

*: p<0.001 vs group PNM group

°: p<0.001 vs AHF group

**Supplemental Table 3.** Comparison of right and left B-lines score according to the PNM site

|  | **Right consolidations**  (n=10; 32%) | **Left consolidations**  (n=8; 26%) | **Bilateral consolidations**  (n=13; 42%) | p-value  among groups |
| --- | --- | --- | --- | --- |
| **Right B-lines score** | 1.66 (1.01 – 4.02) | 1.42 (0.91 – 2.40) | 1.98 (1.20 – 2.80) | 0.75 |
| **Left B-lines score** | 1.65 (1.24 – 3.94) | 1.88 (1.10 – 2.41) | 2.10 (1.20 – 2.90) | 0.80 |
| p-value among groups | 0.91 | 0.72 | 0.84 |  |

Data are presented as n (%), mean and 95% confidence interval if normally distributed, or median and first and third quartile if not normally distributed.

AL: antero-lateral; POST: posterior; AHF: acute heart failure; PNM: patients with only pneumonia; AHF/PNM: patients with acute heart failure and concomitant pneumonia.
